# Supplementary material for: Patterns of Intron Gain and Loss in Fungi
Source: PLoS Biol. 2004 Nov 30;2(12):e422. doi: 10.1371/journal.pbio.0020422 (PMC532390; doi:10.1371/journal.pbio.0020422)
Supplement: Table S1 — Also available at http://genes.mit.edu/NielsenEtAl/. (4.3 MB ZIP). [file pbio.0020422.st001.zip › NielsenEtAl/html/66.html]

AN6186.1.NCU06585.1.MG01699.1.FG05269.1


```
 CLUSTAL W (1.82) Multiple Sequence Alignments - Introns Inserted


Sequence 1: NCU06585.1	1040 aa
Sequence 2: MG01699.1	1045 aa
Sequence 3: FG05269.1	1043 aa
Sequence 4: AN6186.1	941 aa
Alignment Length: 1119 aa
Number Identitical Residues: 277 aa
Alignment Score (without introns) 16530


MG01699.1 	MP---PRKRLRDASLSASANSSSTGNKRQ-KTKLNLP--TVPPRKATLFDSIDTVSTPGA
NCU06585.1	MPPYVPRKRVRDESPPPANDLKNDAKRRKGKENLNAAKLSAPPRKPTLFDALDATASPAS
FG05269.1 	MPPLVPRKRLRESPPPGQGRATKAAKENS----------GASRRKATLYDDLDASATPNS
AN6186.1  	MPPFIRSKRQSSEHNTTSNRHATTAVTTP---------------------SASTSLTPAS
          	**.    **  .   .      . .                           .:  :* :

MG01699.1 	SAKTRSPADILG-VSDEDDSSLSSLSDEEFKDVVAAADRSLDMADEDDDGSDDDDDDIEF
NCU06585.1	SSKTSLAALALDESSDDDSSSLSSLSDGDFEDVPVLKKQKHNVQVGEDE-DEDDDEDLEF
FG05269.1 	NS----VLHGFD-NDDDDGSSLTSLSDDEFEDVVPSKK-----PQGNDD-DSGDDEDIEF
AN6186.1  	DSSLTPVPTDLDNDSSNEDKKVVSDSD------------------------VTDSEEVDW
          	.:.       :.. ..::...: * **                          *.:::::

MG01699.1 	EDVQASLAP---FAEEAAPSGDLELTLDLDGRISLTNEYGNKKGPSKRERITRNAVHRVH
NCU06585.1	EDVEPYQPQSTTANVPEVPAGDLELTLIRDTRISLANALG-KKGPSKLERRIRVSTHCVH
FG05269.1 	EDVAAPLAP---LPDAPVTSGDLELTLTRDTRISLTNAFD-RKGPSKRERKVRHASHCVH
AN6186.1  	EDAIHTAAP---ATSFVSPHENLELTLDRNE-VHLEDILQGQKAPTKIERQIRILIHRLH
          	**.    .          .  :*****  :  : * :   .:*.*:* **  *   * :*

MG01699.1 	VMFLMWHNAVRNSWLCDAEVQGLLLSHVPPRVWEEYERWRRASALDRAS-----------
NCU06585.1	VQLLLWHNAVRNAWLCDPEVQGILLSHLPPKLWDEVDRWRRNSGLEVVVPDGGGTEASTK
FG05269.1 	VMLLLWHNATRNSWLCDPEVQATMISHLTPRLWDEVDRWRRNSGLEKKP-----------
AN6186.1  	VQCLLAHNAIRNDWINDPKCHDILRQRLPPAIQAEIQKWRRASGLEPPE-----------
          	*  *: *** ** *: *.: :  : .::.* :  * ::*** *.*:              

MG01699.1 	-ETPASRSNAPKTKGKGKGSAKETARPSR-RDWGDAAQRLENGTVDMSHGDPLFRLMKAL
NCU06585.1	KKSPAQKKTRAQVKGKAQAKGKDAAKADRSRDWGQAAKHLEKGAVDMSHGDPLFRLMKVL
FG05269.1 	---SPKKPIKDNTKSKGKGK---KVPDRRSRDWGVEAERLEQGAVDMSHGDPLFRLMQSL
AN6186.1  	---APKNPPSQEKKGK-QSK---HQVKSQQRDWGEDSSRQESGQPDLSNGDPIIYLMRIL
          	   ....    : *.* :..        :.****  :.: *.*  *:*:***:: **: *

MG01699.1 	SAWWKQRFRVTAPGLRKVGYMSLERLDRLTKGFKNGDKNTALFGERIGSLDEFRLCAQSC
NCU06585.1	SAWWKQRFRTTAPGLRKWGYMTLERLDRLTKAFNQEEHQPERFGERIASLEDFRECASEC
FG05269.1 	SAWWKQRFRITAPGLRKWGYMSLERLDRLTKAQKAEPHDPEQFGERISDLEGLRHCARIC
AN6186.1  	AAYWKNKFKITAPGLRKHGYRPMSQLQTQIASFHKDKHDPELYGEKIPSVEEFRQAAERM
          	:*:**::*: ******* ** .:.:*:    . :   ::.  :**:* .:: :* .*   

MG01699.1 	TGSRDVGAQLFTALLRGLGLEARMIASLQ1PLGYGWSKYEDADPEEERSS---QPSPEKP
NCU06585.1	AGSRDVGAQLFTAMLRALGLEARLVASLQ~PLGFGWTKVEEADPEKEEDL---KPTPKKN
FG05269.1 	EGSRDVGAQLFTALLRGLGLEARMVTNLP~CLGFGWNKLEEAEPEKSDSTNQTKATPDKK
AN6186.1  	EGSRDLGAQLFTALLRAIAIEARLVASLQ~PLGFGWTKAEAHT------------SPKSK
          	 ****:*******:**.:.:***:::.*   **:**.* *               :*.. 

MG01699.1 	TQTTQTPQKNTKNEPRRQHVSSRSRGKQSKAIEEEDSNYVDDFEPQEVNSDDEMVVEVPK
NCU06585.1	MGATSMAKETVTTHASG---RSTRRGASRKATKIQRSN----SELIAADSDDDLVISDHE
FG05269.1 	TETTKKTAATTKKQSSKKTIQAAKNTTRKAKSKPKVASDSEDLELEYKDTDDESVVDMEV
AN6186.1  	TKPKHTNLESDGD--------------------------------GFPDSDEEEDAQPSS
          	  ..     .                                      ::*::   .   

MG01699.1 	KMAP---QSKKFDQDLEFPHYWTEVLSPVTHKYLAVDPVVKF-VIATNRELVESLEPRGS
NCU06585.1	PEPTPRMKNKVYDKDLEFPHYWTEVLSPVTKKYLPVDPIVKS-IIGTNRELIESLEPRGS
FG05269.1 	TPRK--ISTKKYDADMDFPHYWTEVLSPVTNKYLPVDAIVKN-VVGTNRDLIESLEPRGA
AN6186.1  	RGRK---AAPRYDQDLPFPIYWTEVVSPITHQVISVDPLVLSNAVAATQELQAAFEPRGA
          	           :* *: ** *****:**:*:: :.**.:*  . :.:.::*  ::****:

MG01699.1 	RTEKARQVMAYIMGFSSDGTAKDVTVRYLKRQMLPGRTKGVRYPIEKVPVYNHKGKVKHY
NCU06585.1	KADKAKQVMAYVMGYSPDGTAKDVTVRYLKRQTLPGRTKGMRIPMEKVPVYNKHGKVARY
FG05269.1 	KADKARQIMAYIIGYSQDGTAKDVTVRYLKRNMLPGRTKGVRMTPEKVPVYNRHGKIKRY
AN6186.1  	KAEKAKQVICYVIAFSADKTAKDVTTRYLRRRTWPGKTKGFRLGKKGP----------DD
          	:::**:*::.*::.:* * ******.***:*.  **:***.*   :              

MG01699.1 	EHVDWIKSVLRGYVRGNKRHP-ITEVDEEEDSTDLRPAKHEK-KEVKEGDETLQYYKQSK
NCU06585.1	EMYDWFQTVMKGYARGGRSKPAMTDVDLEENATDLKPAKPEK-KEVKEGQETLQYYKQSK
FG05269.1 	DQFDWFKTAISGYRRGSKNHP-ITEIDEAEEATDLKPAKPEK-KEVKEGQETLQYYKQSK
AN6186.1  	DLLDWFRVLLRNYERPYKDRT---AVDDIEDAKDLVPNRPTKSKPTNETVDTLQSLRTSS
          	:  **::  : .* *  : :.    :*  *::.** * :  *:* .:*  :***  : *.

MG01699.1 	EYVLERHLKREEALLQDATPVKVFKVKAKGG------------EFTEENVYLRRDVVQVK
NCU06585.1	EFVLERHLKREEALLPNARPVKMFRNKGSSGGSSKK-AATTTAATQDEPVYSRKDVVHVK
FG05269.1 	EFVLERHLKREEALNPEAKPAKVFKNKGKGG------------KVEEEDVFLRSDVLNVK
AN6186.1  	EFVLERFLRREEALRPGALPVRTFTPGGKKKNANGNGASTPTESPKAENVYRRSDVVKCQ
          	*:****.*:*****   * *.: *   ..  .:. ..:::.:     * *: * **:: :

MG01699.1 	SAETWHKQGRAPKEGEKPLKMVPYRAATMNRKRDIAAAEAATGKKVLQGLYSMDQTDWII
NCU06585.1	SAETWHKQGRAPLPGEQPLKRVPYRAATNNRRREIAEAERLTGQKVLQGLYSFDQTDWII
FG05269.1 	SAETWHKQGRAPKPGEQPLKKVPYRAATLNRKREIMEAEAATGAKVLQGLYSWDQTEWII
AN6186.1  	TAESWHKEGREPLPSAKPLKHVPIRAVTLLRKREVDEEARRTGQKPLQGLYSFEQTQEII
          	:**:***:** *  . :*** ** **.*  *:*::      ** * ****** :**: **

MG01699.1 	PPPIKDGIIPKNEYG~NIDLFAEHMCPQGAVHVPFRGAVKVCRRLGVDYAEAVIDFEFGH
NCU06585.1	PPPIENGVIPKNEYG~NIDLFAEHMCPQGAVHVPYRGAMRVCKRLGIDYAEAVVDFEFGN
FG05269.1 	PPPIKDGVIPKNDYG2NIDLFAEHMCPKGAVHVPFRGAIRVCKKLQIDYAEAVVDFEFGH
AN6186.1  	PPPIVDGIIPKNEYG~NIDCFVPRMVPKGAVHIPFSGTARICKKLGIDYAEAVTGFEFGS
          	**** :*:****:** *** *. :* *:****:*: *: ::*::* :****** .**** 

MG01699.1 	RMAVPVIQGVVIAEEHHDRVMEELAKDEAERARKEDAKRTAAALAMWRKMLMAMRITNRL
NCU06585.1	RMAVPVVQGVVIAEEHHDQVMEEVAKDEAEKKRKEDEKRRKAALGMWRKLLMGMRIVERI
FG05269.1 	RMAVPVIQGVVIAEENHDMVMVELEKDEAERARKEDEKRRKKALAQWRRFLMGMRIAERI
AN6186.1  	QMAVPVIEGVVVAAENKDLVVDAWRADNEEKRRKEARKAEAKILATWRKFLFGLRIAQRV
          	:*****::***:* *::* *:     *: *: ***  *     *. **::*:.:**.:*:

MG01699.1 	REEYGNVGDGDLRIIQTSRSRADETTHRPADASAGFDEDTAGG-FLPAGYECESQETEDK
NCU06585.1	KQDYGHVGDEVEVFGRHGSGGADIRELSDAEAEMGEDEETGGGGFLPEGFEVEDEERDSH
FG05269.1 	RQEYGEITDDISVFG-----HARDSAHSKKPPSVP-DEDMAGG-FLPEGYEEEVEEDQEV
AN6186.1  	QEEYAEDEEGHLPDAHNPFASRKASTHARGHTQTPAEEQHPAG--LVDEEEEEEEADR--
          	:::*..  :          .           ..   :*:  .*  *    * * :     

MG01699.1 	AVENQEAPRVSSYFPVAGEDDEDDD------DDLVVEDAQMDDATSPALAGGSAIIGDTE
NCU06585.1	LR--------SSFFPVVDEDDEDGE------DTLEVDHG---------------------
FG05269.1 	PVHHT-----SSFFPAVDEDDEGDDGLVMEHDGVDQQRAMVQMDINAEEAAPFKSQAEAG
AN6186.1  	----------GGGFLLGDDHADDGD------LIIEVRQPPVRRVQN--------------
          	          .. *   .:. :..:        :           .              

MG01699.1 	RSGEHGDALASDGEQHQADAP---VMEEDAVYS----NGSSRSAASKR-----RRATVEA
NCU06585.1	-----------EGEGNGDQAE---RKKESAVMS----TKPKRKAAAAK-----AKASIPG
FG05269.1 	RDPESKAELECEMDQDTEQEEPKLELESDAEFEQPRTSARSRKAAPKKQTKKERQPPLRA
AN6186.1  	--------ISSENESLSGDVGRGGGFLLDGNND----SNDEGQLAQMR-----KPPVRRI
          	          .: :    :         ..  .    .  . . *  :       .    

MG01699.1 	SPATRVR--PSRQ---AAKKAKPKLKQLESDIDTQ------
NCU06585.1	SRRRTRRNVPSSEDEDEEEDEDSELSDLEDDEEDF------
FG05269.1 	TRRSTRSGRKVVSYGEDVVHNDDEIADSYAESEDDE-----
AN6186.1  	PRILYRDGSPVLEEESSPEEYTEDISPPPQKRRRTRNSRKT
          	.      .    .  .   .   .:     .     .: .:
```
